# Detection of centroblast cells in H&E stained whole slide image based on object detection

**DOI:** 10.3389/fmed.2024.1303982

**Published:** 2024-02-07

**Authors:** Sumeth Yuenyong, Paisarn Boonsakan, Supasan Sripodok, Peti Thuwajit, Komgrid Charngkaew, Ananya Pongpaibul, Napat Angkathunyakul, Narit Hnoohom, Chanitra Thuwajit

**Affiliations:** ^1^Department of Computer Engineering, Faculty of Engineering, Mahidol University, Nakhon Pathom, Thailand; ^2^Department of Pathology, Faculty of Medicine Ramathibodi Hospital, Mahidol University, Bangkok, Thailand; ^3^Department of Pathology, Faculty of Medicine Siriraj Hospital, Mahidol University, Bangkok, Thailand; ^4^Department of Immunology, Faculty of Medicine Siriraj Hospital, Mahidol University, Bangkok, Thailand; ^5^Image Information and Intelligence Laboratory, Department of Computer Engineering, Faculty of Engineering, Mahidol University, Nakhon Pathom, Thailand

**Keywords:** H&E, whole slide image, object detection, centroblast, artificial intelligence

## Abstract

**Introduction:**

Detection and counting of Centroblast cells (CB) in hematoxylin & eosin (H&E) stained whole slide image (WSI) is an important workflow in grading Lymphoma. Each high power field (HPF) patch of a WSI is inspected for the number of CB cells and compared with the World Health Organization (WHO) guideline that organizes lymphoma into 3 grades. Spotting and counting CBs is time-consuming and labor intensive. Moreover, there is often disagreement between different readers, and even a single reader may not be able to perform consistently due to many factors.

**Method:**

We propose an artificial intelligence system that can scan patches from a WSI and detect CBs automatically. The AI system works on the principle of object detection, where the CB is the single class of object of interest. We trained the AI model on 1,669 example instances of CBs that originate from WSI of 5 different patients. The data was split 80%/20% for training and validation respectively.

**Result:**

The best performance was from YOLOv5x6 model that used the preprocessed CB dataset achieved precision of 0.808, recall of 0.776, mAP at 0.5 IoU of 0.800 and overall mAP of 0.647.

**Discussion:**

The results show that centroblast cells can be detected in WSI with relatively high precision and recall.

## 1 Introduction and background

Follicular lymphoma (FL) is the second most common lymphoid malignancy both in Western and Asian countries. It accounts for about 5–35% of non-Hodgkin lymphoma (1–3). The majority of FL harbors *t*_(14;18)_, resulting in overexpression of the BCL-2 protein. In general, patients with FL are present with lymphadenopathy and infrequent B-symptoms. A combination of clinical and laboratory findings, as well as the histopathological grade of the disease, can help predict the course of the disease. Some patients may have a relatively indolent course or a slow progression and can live for many years without any treatment, while others may have a more aggressive course and a shorter survival time without timely and properly treated. The diagnosis of FL is usually established by a histopathological examination together with immunohistochemical studies.

The histological grade is determined by the number of the enlarged B cell with multiple eccentric nucleoli and high proliferative capacity in the germinal center of secondary lymphoid follicle known as centroblasts (CBs) presented in a tissue sample. At present, the World Health Organization (WHO) classification system is the standard for FL grading. The WHO classification system is used to grade FL into three categories: grade I (0–5 CBs per high-power field), grade II (6–15 CBs per high-power field), and grade III (>15 CBs per high-power field). Grade III is further divided into grades IIIa and IIIb. In grade IIIa, a few centrocytes are found mixed with the CBs; however, only pure populations of CBs without admixed centrocytes are seen in grade IIIb. Grades I and II are considered low risk and may not require treatment unless the patient has problematic symptoms, while grades IIIa and IIIb are considered high risk and often require chemotherapy (2). Currently, the process of grading FL involves manually counting the CBs in tissue samples using a microscope in conventional microscopic high-power fields (HPFs) stained with hematoxylin and eosin (H&E). Traditionally, the average CB count from 10 HPFs is used for grading.

As the number of CBs affects the grade of the disease, which is considered one of the prognostic factors, evaluation and CB counting are essential in the diagnostic process. However, the current routine practice is time-consuming and prone to subjectivity and variability. Tumor heterogeneity may produce a selection bias during the process. This results in high inter- and intra-observer variability and is vulnerable to sampling bias. Indeed, inter- and intra-pathologist variability has been reported, which normally ranges from 61 to 73% (4). As the CB counting process is subjective, not every pathologist will be able to provide reproducible results. All these issues directly affect the clinical management of patients. Hence, improving the reliability and reproducibility of histological grading is of great importance.

Hence, the enumeration of CBs is important to determine the proper treatment. Based on the manual reading of H&E and immunohistochemical staining slides by pathologists, the process takes time and some mistakes can occur. Moreover, CBs are often confused by similar-looking cells within the tissue, therefore a system to help their classification is necessary.

In this study, we focus on the detection of CBs in HPF patches to count the number of CB cells automatically. The region(s) of a WSI from which the patches are extracted from were chosen by manual inspection at low magnification.

## 2 Materials and methods

### 2.1 H&E staining of FL and patients information

This study included 88 cases of FL admitted for treatment at the Faculty of Medicine Siriraj Hospital between 2016 and 2020. The protocols for collecting and using tissue were approved by the Siriraj Institutional Review Board (SIRB) (COA no. Si973/2020). Detailed information about the patient group is shown in Table 1. Formalin-fixed paraffin-embedded (FFPE) tissue samples were prepared for automated H&E staining at the Department of Pathology, Faculty of Medicine Siriraj Hospital, Mahidol University, and scanned at a resolution of 0.12 μm/pixel using a 3DHistech Panoramic 1,000 microscope with a 40x objective lens. The resulting images were saved in mrxs format for further analysis.

**Table 1:** Demographic data of FL patients and clinicopathological correlation.

**Characteristics**	***n* (%)**
**Age (year) (*n* = 88)**	
≥60	56 (64.0)
<60	32 (36.0)
**Sex (*****n*** **= 88)**	
Female	44 (50.0)
Male	44 (50.0)
**Grading (*****n*** **= 87)**	
1	49 (56.3)
2	21 (24.2)
3	2 (2.3)
3a	12 (13.8)
3b	3 (3.4)
**Hemoglobin level (*****n*** **= 86)**	
<120 g/L	86 (100.0)
≥120 g/L	0 (0.0)
**No. of nodal areas involved (*****n*** **= 84)**	
≤ 4	35 (41.7)
>4	49 (58.3)
**Serum lactate dehydrogenase (*****n*** **= 84)**	
≤ 280 U/L	33 (39.3)
>280 U/L	51 (60.7)

### 2.2 Machine learning background and related works

In early works that focused on applying image processing and machine learning to CBs, it was considered an image classification problem—deciding whether a cell is a CB or not, given a single-cell image that had already been cropped, manually or otherwise. Some studies also addressed the segmentation of individual cells from HPF images, typically by thresholding and image morphological operations. In (5), 41 and 53 images of CBs and non-CBs (each showing a single cell) were obtained from 11 HPF images. Features based on pixel homogeneity as well as histogram statistical features were extracted from each image. Principal component analysis (PCA) was used to reduce the size of the feature space, and the *k*-nearest neighbor classifier was used. In (6), the authors address segmentation by Otsu thresholding followed by morphological image operations. The classification features were obtained by transforming each cell image to the frequency domain and performing PCA on the spectral features. The reported accuracy was 82.56% over the size of the test set of 88 images. In (7), which was a continuation of (5) by the same group, the study was extended by new features, larger dataset size, and *k*-fold validation during training.

Some studies addressed the problem of where to extract the HPF patches. One strategy was to make use of not only H&E-stained WSI but also the immunohistochemical (IHC) stains such as the CD10/CD20 of the same tissue sample. Tumor regions are more clearly discernible under IHC stains and can be used to help guide where to zoom in. The disadvantage, besides needing more laboratory procedures and resources, is that the two WSIs now have to be registered together before the additional information from the IHC stain can be utilized. In (8), H&E and CD20 WSIs were registered together, where the objective was to classify each HPF as one of the three classes: mantle, follicle, and intrafollicle. The follicle is the region where one would want to be searching for CBs. The authors used standard image features such as histogram statistics, with support vector machine (SVM) and *k*-nearest-neighbor (KNN) as the classifiers, and reported accuracy of around 60%.

Several more studies later on proposed new features for the classification of CBs. The studies (9, 10) focused on the classification of CBs without segmentation. The approaches are similar except for the feature engineering, which were a combination of color and morphological and frequency domain statistics. Heavy feature engineering was very common in the machine learning field before the rise of deep learning. Both studies obtained ground truth labels using consensus of multiple pathologists and reported similar classification accuracy in the 80% range. Later, Kornaropoulos et al. (11) proposed a novel feature based on the concept of subspace from signal processing. Cell images are flattened into vectors, which form columns of a rectangular matrix. The matrix is then decomposed using singular value decomposition (SVD). Subspaces of the positive (CB) and negative (non-CB) were constructed using the top *k* largest singular values and by projecting each original vector into its class's subspace. At inference time, a query vector is classified by solving a least squares problem to find the centroid that the vector is closer to. The study reported very high classification accuracy of around 99%. The dataset size was 213 CB and 234 non-CB. The disadvantage of this method is that it requires solving the SVD of a matrix of size #pixels by #training examples, which does not scale to a large number of examples. The same team shortly after published (12), which proposed a complete pipeline including preprocessing and segmentation. The segmentation part followed standard image processing techniques as in earlier research. Average recall value for CBs over three test WSIs was 82.52%. This shows that high classification accuracy alone does not transfer over to high detection performance, since any segmentation error will impact the downstream classifier that was trained only on perfectly segmented cell images.

A larger scale study for CB classification was proposed in (13), involving 3,771 and 4,000 CBs and non-CBs, respectively. The feature extraction and classifier were standard techniques as used in other previous studies. Segmentation was performed by image registration with CD20-stained WSIs, which provides much better follicle delineation than using H&E stain alone. The classification accuracy was reported at 80%.

### 2.3 Deep learning approach to WSI classification

Studies after this point were after deep learning (DL) (14) had become popular and the focus shifted from feature engineering to applying DL to entire WSI classification, not individual cells. For example, this can be used to classify WSIs into different stages or types of cancer. DL is very successful at image classification, matching, or surpassing humans on many such problems, and it is logical to consider applying DL to WSI classification. Unfortunately, unlike standard image classification, where the images can be resized to a size that can be accommodated by a deep neural network, typically around 224 × 224 up until 1, 024 × 1, 024—resizing huge WSIs to such small sizes will result in the loss of all details. Feeding an entire WSI into a deep neural network and training it the same way as a standard image recognition problem, while simple in principle, cannot be done in practice since it would require much more memory than any graphical processing unit (GPU) has at present. While this feat was recently achieved by Chen et al. (15) by taking advantage of CUDA (a GPU programming library) unified memory architecture, it took 2 months to train the model on a supercomputer, thus impractical for most research teams. It should also be noted that the size of the WSIs used in this study is 10, 000 × 10, 000 which is still relatively small, as WSIs of size 50, 000 × 50, 000 or more are not uncommon.

While the results of many studies were promising, image classification where the features are hand-crafted are known to be brittle—the performance rapidly decrease with small deviations from the statistics of the original training data. Moreover, the feature engineering must be performed each time a condition is changed, such as a new WSI imaging machine.

Because an entire WSI will not fit in GPU memory, or even the system memory of most computers when fully decompressed, applying DL to WSIs means that one must operate at the patch level rather than at the slide level. WSIs utilize hierarchical representation that trade size for resolution, and when viewed at full size (the entire slide is visible on the screen), the resolution (px/μ*m*) is the lowest. When viewed at full resolution, the situation is reversed and one screen shows only a tiny fraction of the slide. The patch level refers to images cropped from the slide zoomed in all the way, i.e., at full resolution. One can crop at this level and choose the patch size that current hardware is able to handle. The problem is then how to make a decision for the entire slide while looking only at just the patches.

The approach to solve this is to aggregate the decisions made by a patch level model on a group of patches that are chosen as the representative of the entire slide. The patches are chosen by hand, then a model classifies each patch and then the results are converted into a slide-level decision. This can be as simple as by voting or averaging of the class probabilities, or another model can be trained to combine the outputs of the patch level model into a slide level label. This is called the patch level model approach in the literature.

The patch level model approach can be improved by using multiple instance learning (MIL), first proposed in the context of WSI classification by Hou et al. (16). MIL first divides the slide into regions (chosen manually or otherwise) called bags. A bag is considered negative if all the patches inside of it are classified as negative by a patch level model. On the other hand, a bag is considered positive if at least one patch inside of it is classified as positive. MIL then proceeds in two phases; First, is patch selection, where *k*-most positive patches are chosen from the positive bags. These *k* patches are the “representative” of the entire positive slide and constitute a “positive example” for the slide-level model. A negative example is obtained by randomly sampling from the negative bags. The slide-level model is then updated like in a standard binary classification problem. The main advantage of MIL over the standard patch level approach is that no patch level labeling by hand is needed for new WSIs, at least once a patch level model is available. All patches are selected during training and are assumed to be the same class as what the patch level model predicts, an assumption called weak supervision in the literature. A large-scale study using MIL for WSI classification was done in (17), involving thousands of slides in each cancer category.

Although successful, MIL is basically a workaround. It also brings new challenges. The first is that the ranking of the *k* top patches are done using outputs of the patch level image classification model. One of the common misconceptions in DL is that the output probability indicates “how sure” the model is given a particular input, i.e., an output of 0.2, 0.8 is more sure that the answer is class 1 than the output of 0.4 and 0.6. This has been shown to be not true (18), and there is no way to rank the confidence of the model's output without spending a significant amount of computation doing variational inference. The second challenge is selecting the value for *k*. If it is too low, the number of informative (positive) patches may be less than what it actually is, and on the other hand, if it is too high, then irrelevant (negative) patches may be included in a positive training example. For simplicity, most studies set the value of *k* = 1 to take the most relevant patch only from each bag, but this is complicated by the problem stated earlier that there is no easy way to rigorously rank the patches. Finally, using MIL involves having two models that operate at different levels and there is no way to train both of them together in an end-to-end manner because of the sampling operation between the patch level model and the slide level model.

For these reasons, we propose the use of object detection as a patch level (and the only) model. We assume in this study that the regions of interest at the slide level have been previously selected by a pathologist or obtained a priori by other means. Alternatively, all non-background patches in a WSI can be considered the region of interest—an approach that requires more bootstrap labeling for the training the initial patch level model. The advantage/disadvantage of using object detection instead of MIL can be summarized as follows:

1. It is conceptually the same task as what a human pathologist does, which is to detect and count the number of CBs for each HPF. There is no need to train a separate slide level model for aggregation because the number of CBs is already what is needed in clinical practice.
2. The object detection model that we used is engineered for fast inference, as it is common for this kind of model to be applied to real time detection in streaming video. In contrast, image recognition models are not designed with fast inference being a priority. Inference speed is especially important if the entire non-background area of the WSI is considered as the region of interest, as thousands of tiles must be processed for one WSI.
3. The disadvantage is that this approach is only applicable to lymphoma since it focuses exclusively on CBs.

### 2.4 Object detection

The object detection task is to draw a tight bounding box(es) around the object(s) of interest in an image. For the current use case, we are interested in its ability to count the number of objects (CBs) in a patch. There are currently three different families of object detection models in wide use: RCNN (19), SSD (20), and YOLO (21). We choose the YOLO family for this study due to its unique combination of fast inference speed and detection accuracy. YOLO is a single-pass object detector that is designed to be trained end-to-end from the beginning. In the original version, an image is divided into a grid of 11-by-11 cells. Each cell is responsible for detecting at most two objects whose centroid's coordinates fall within the cell. The target output of the model is a 3D array of shape 11-by-11 by L. The class label and bounding box coordinate of each object (or two objects) is encoded in the 1 by L vector “tube”. In addition, part of the tube is the probability that this cell has an object, set to 1 for cells that actually contain an object and 0 for cells that do not. The loss function is a weighted sum of softmax loss for object type classification, the mean square error for the bounding box coordinates, and the “objectness” probability. Later versions offered improvements such as normalizing the bounding box coordinate, anchor boxes, increased input resolution, and speed optimization. Recently, there are multiple groups that claim to be the latest official version. We choose the version 5 implementation at https://github.com/ultralytics/yolov5 since the repository is highly active with over 200 contributors and almost 30 thousand stars on Github. There is no associated publication for version 5. The last published article was for version 4 (22).

### 2.5 Data collection

The HPF patches for model training and validation were obtained by a two-phase process. The challenge was that having pathologists inspect entire slides to search for all CBs is impractical, because each WSI can have hundreds of thousand of patches, most of which do not contain CBs. Therefore, to create the training data, we first cut each WSI into individual patches and then applied the Hovernet cell segmentation model (23) to each patch. The segmented cells were then filtered with the following heuristics to detect potential CBs: each segmented cell's area was calculated using contours, and this gives the area in pixel squared. We made the assumption that each cell's shape is approximately a circle and calculated the diameter of the circle that would have the same area. Then, the unit of the diameter of the circle was converted from pixels to microns by the conversion factor of 0.12 μ*m*/pixel (the resolution of our equipment). The diameter was then used as the selection criteria, and we included cells which have an equivalent diameter of at least 5.13 μ*m* and an aspect ratio (the ratio between the major and minor axis of the cell's contour) between 0.7 and 1.3. These criteria are based on the general characteristic of CBs that they tend to be large and approximately round. Cells that match these criteria are considered as potential CB, and the patch that contains the cell(s) was saved for data labeling by pathologists, who see just the raw patches without any prior labels or annotation. Due to the labor-intensiveness of the labeling process, we were not able to use all the patches from all WSIs.

**Figure 1:**
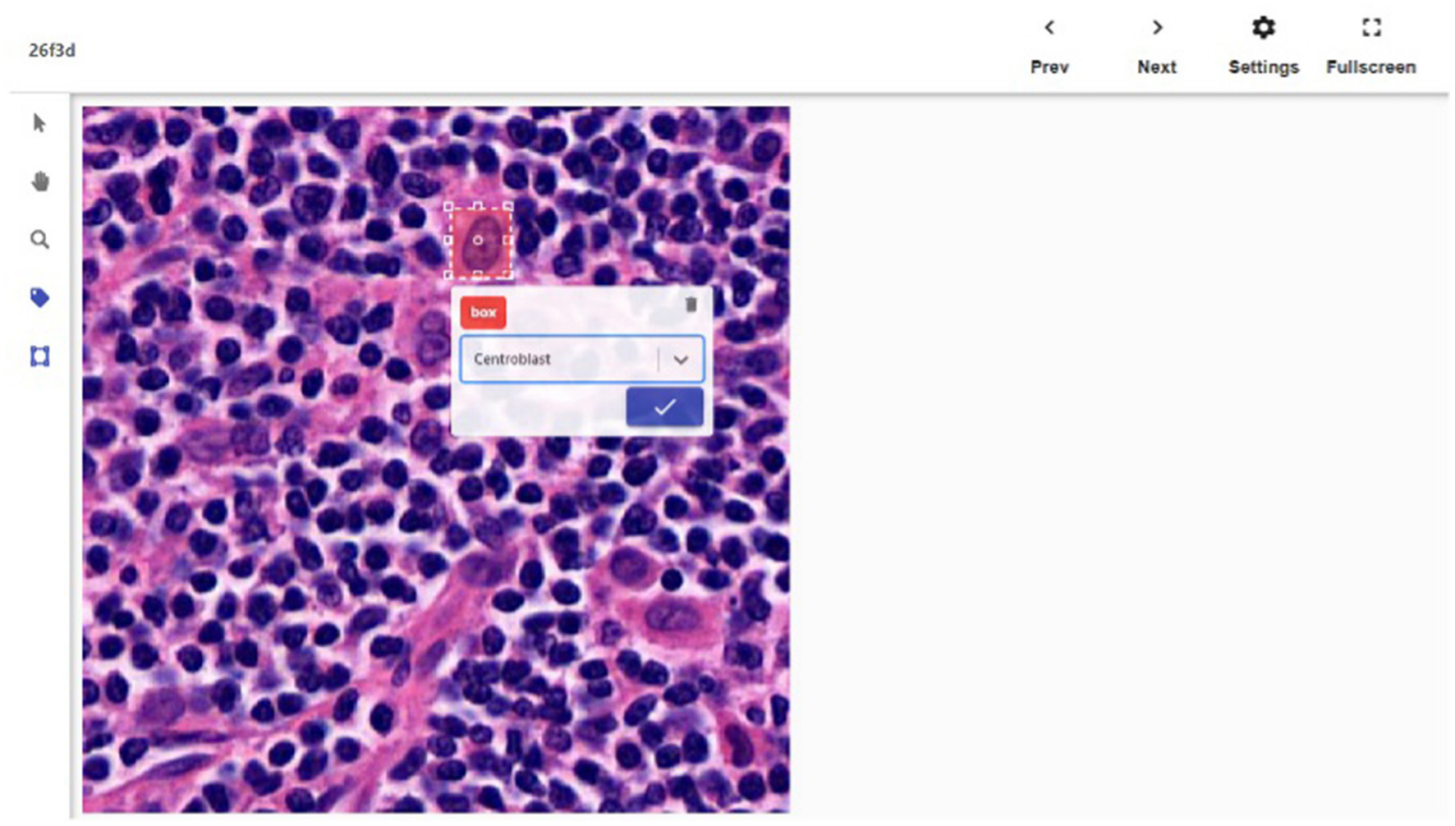
The detail screen of one of the patches. The bounding box is shown being drawn.

We created a web-based tool (Figure 1) that allows multiple pathologists to work on data labeling in parallel and prevent two pathologists from working on the same image. There are two types of users in this system: labelers and reviewers. A labeler can draw bounding box(es) around CB(s) in a patch, or just click confirm to pass if the patch is a false positive and does not actually contain a CB. A reviewer can sign off a patch and also make modification to the bounding box(es) if necessary. We only include patches and bounding boxes that had both been labeled and reviewed in the training data.

As mentioned, we gathered training data in two phases. In the first phase, candidate patches were identified using pre-trained Hovernet and the heuristics as mentioned above. After labeling and validation, we obtained 445 instances of CB bounding boxes that could be used to train the first generation object detection model. We then used that model to scan through another set of patches. Since the first generation model is already trained to detect CBs directly, the second phase no longer need the size/aspect ration heuristic, and the patches where CB(s) were detected were saved for the second round of labeling. In total, both phase combined gave a total of 1,669 instances of validated CBs instances for (second generation) model training.

### 2.6 Model training

We trained the YOLOv5 object detection model on the dataset with 1,669 CB instances for the training set and the rest for the test set. Unless stated otherwise, the training hyperparameters are the same as in defaults in the file data/hyps/hyp.scratch-low.yaml under the Github repository https://github.com/ultralytics/yolov5. The other hyperparameters not included in the file are as follows: batch size = 16, number of epochs = 100, and the optimizer used was SGD (stochastic gradient descent). The training environment was Ubuntu version 20.04 with the GTX2060 as the GPU. The input image size, model size, and preprocessing were varied during the experiments. YOLOv5 offers several different model sizes, and we experimented with sizes m (smallest), x and x6 (largest).

### 2.7 Experimental setups

**Figure 2:**
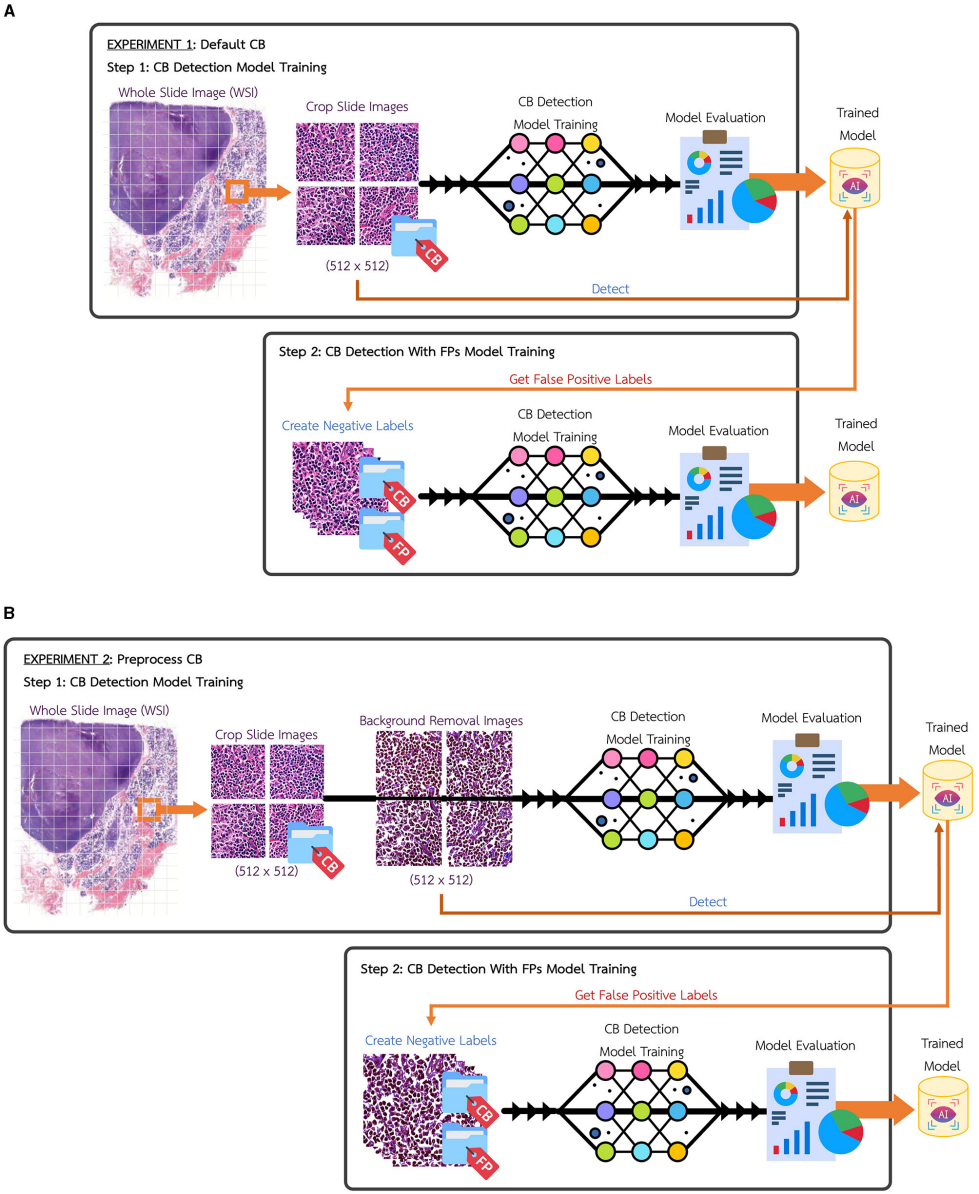
**(A)** Experimental setup of Experiment 1, default CB. **(B)** Experimental setup of Experiment 2, preprocess CB.

In this research, the whole slide image (WSI) for Centroblast (CB) dataset have been collected and slices into 512 × 512 pixels tiles images. After labeling and validation were performed, the total dataset gave 1,669 instances of validated CBs from 1,205 tile images, as illustrated in Table 1. Figure 2A shows the experimental setup using the default CB dataset, named Experimental 1 Default CB. In the first step (Exp1-1), the dataset is fed into detection model, YOLOv5, and the best model is evaluated and selected. The selected model is used for collecting false positive instances, in other terms, the non-CB objects, in the second step (Exp1-2). The non-CBs instances is created and added to default CB dataset, the new dataset now containing 2 classes, the CBs and non-CBs.

**Figure 3:**
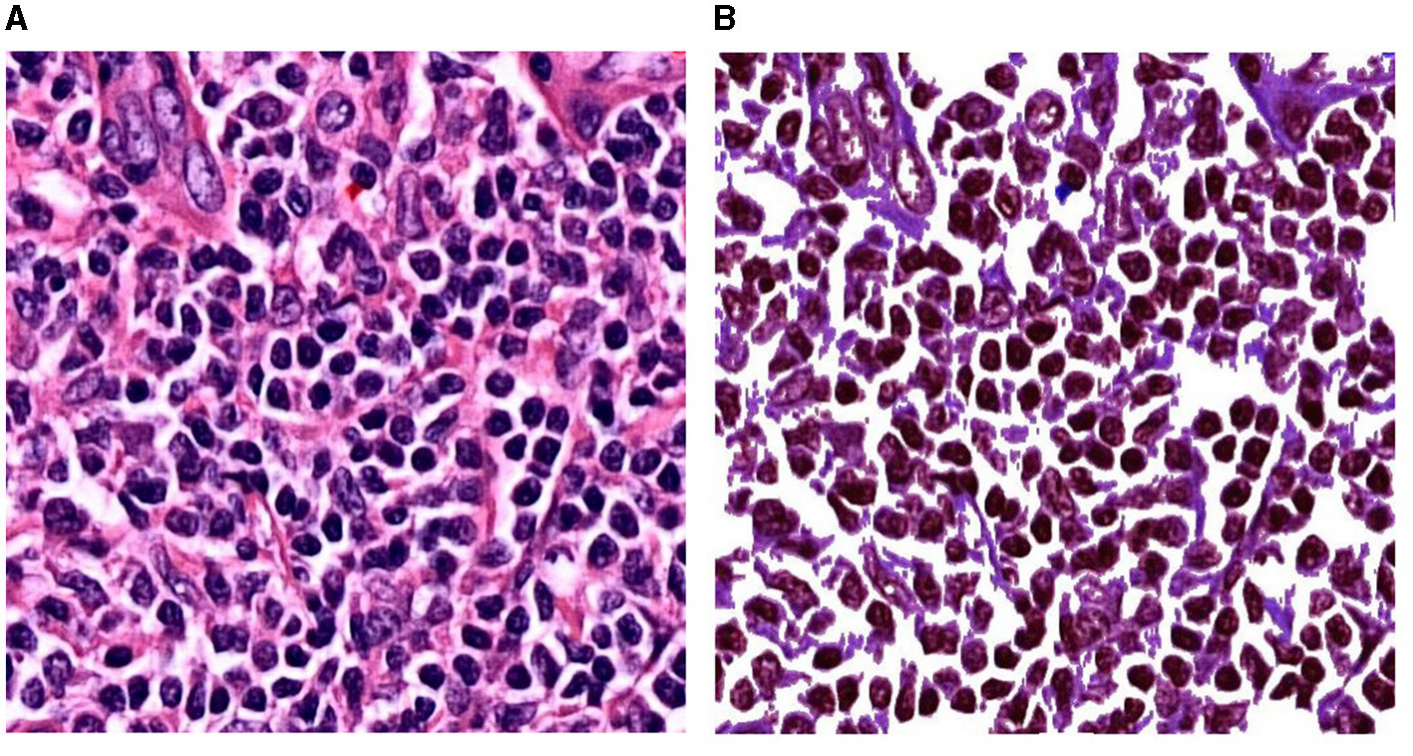
Experimental setup of Experiment 2. **(A)** A patch before preprocessing. **(B)** The same patch after preprocessing.

Figure 2B shows another experimental setup using the preprocess CB dataset, named Experimental 2 Preprocess CB. The default CB dataset undergos preprocess method for creating clear CB instances. The histogram equalization is used to adjust intensity, brightness, and channel of the image. Then, image contour is used to eliminate overly large area and tiny noise in the images. As the images contain similar shades of purple, the cells are mostly visible in darker shade of purple. The light purple color is adjusted to white, where it is higher than average color value of the whole image. Figure 3 illustrates the differences of (a) default CB datasets to (b) preprocess CB datasets. In the first step (Exp2-1), the preprocess dataset then feed into detection model, YOLOv5, and the best model is evaluated and selected. The selected model is used for collecting false positive instances, in other terms, the non-CB objects, in the second step (Exp2-2). The non-CBs instances is created and added to preprocess CB dataset, the new dataset now containing 2 classes, the CBs and non-CBs.

**Figure 4:**
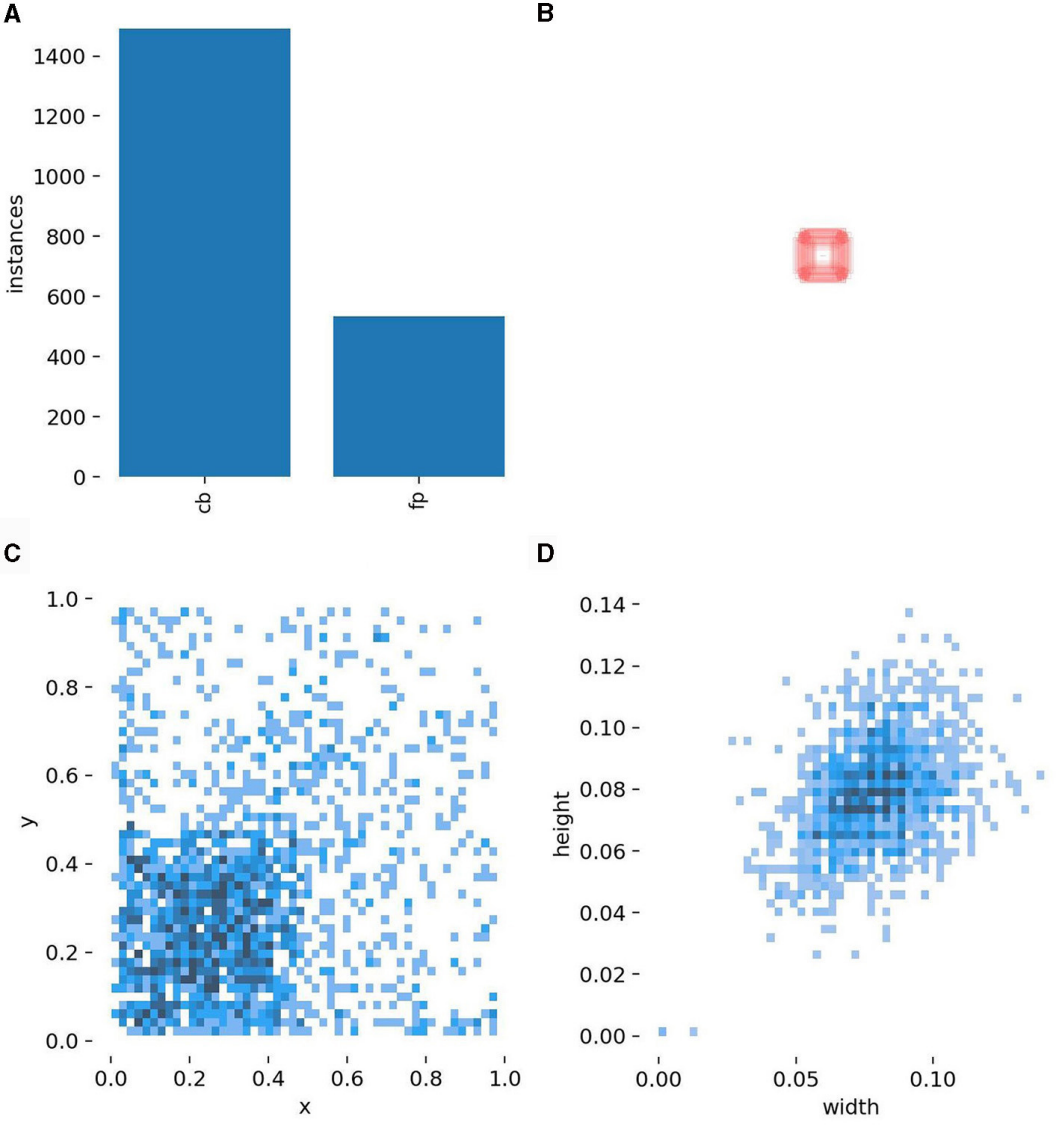
Descriptions of the CB data. **(A)** The number of actual CB cells vs. false positives (negative examples). **(B)** The sizes of the bounding boxes. **(C)** The distribution of the position within a patch of CB bounding boxes. **(D)** The distribution of the weight and height of CB bounding boxes.

After performing the mentioned experiments, the total dataset of 1,669 CB dataset is containing 1,205 images with CBs instances and 629 non-CBs instances in both experiments. Table 2 shows the partition of dataset for training YOLOv5 detection model. The test dataset is separated by 5% of total dataset, while train and validation dataset are divided by 80–20% from the rest. The non-CB labels are collected by controlling the number of instances to not more than CBs instance and controlling its shape to CB's similarity. Figure 4 shows illustration of CBs and non-CBs instances, where (a) shows plot of instances, (b) shows size of bounding boxes of CBs and non-CBs instances, (c) shows distribution of bounding boxes from overall dataset, and (d) shows distribution of size of bounding boxes from overall dataset.

**Table 2:** Dataset information.

**Dataset**	**Train**	**Validate**	**Test**	**Total**
Images	915	229	61	1,205
CB labels	1,275	313	81	1,669
Non-CB labels	517	96	16	629

### 2.8 Performance measurement of object detection

Performance of object detection is measured by a quantity called mean average precision (mAP). A predicted bounding box is considered a true positive (TP) if the intersection over union between the predicted bounding box and the ground truth bounding box is over a certain threshold, typically 0.5. The IOU has its maximum value at 1.0 when the predicted box and the ground truth box perfectly overlap. On the other extreme, the minimum value of IOU is 0 when the predicted box and the ground truth box do not overlap at all. A predicted box is considered a TP if the IOU between it and the corresponding ground truth box is ≥0.5. If a predicted box gives IOU less than this value either because it is in the wrong location or because the corresponding ground truth box does not exist, it is considered a false positive (FP). Once the value of TP and FP are obtained, the precision can be calculated by the standard formula $\text{precision}=\frac{TP}{TP+FP}$. The precision value is calculated once for each confident threshold—a model will predict a box when its confident is above this value. By varying the confident threshold value and calculating the precision for each one and then taking the average, one can get the average precision (AP) value. Finally, the AP values for each class of object are averaged to get the final mAP value. In this study, since there is only one class of object—the CB—the AP value is the same as the mAP value. In the results, mAP@0.5 means that the mAP value was calculated using IOU threshold of 0.5. While mAP with no @ is the average of mAP values calculated at different IOU thresholds—typically 0.5–0.95 in steps of 0.05. For context, mAP value of around 0.5 (50%) is considered high, see for example Figure 1 of (22).

### 2.9 Image preprocessing to boost the performance of object detection

Due to the fact that the WSIs in this study were collected retrospectively from existing images, some were scans of slides that were quite old. The quality of these WSIs is not homogenous. We observed that generally the object detection result was better on newer WSIs that are purple color, where the cell images appear to be slightly clearer than older slides with pink color. To boost the performance of the object detection algorithm, we decided that the best approach was to improve the clarity of the input patch images by removing the non-cell background as much as possible. To this end, we introduced preprocessing steps that can be outlined generally as follows:

1. Given an input patch image, perform histogram equalization.
2. Convert the histogram-equalized image to grayscale.
3. Calculate binary image from the grayscale image using Otsu's method (24).
4. Perform contour detection on the binary image.
5. Calculate the average area of the rectangular bounding box of the contour of each cell
6. Perform the dilation operation on the binary image.
7. For each contour whose area is less than half of the average area, “fill in” the binary image by drawing solid lines over the contour lines.
8. Use the processes binary image as a mask to select the foreground pixel from the original patch image, and fill all the unselected pixel with white (pixel value 255, 255, 255) color. This final image becomes the input to the object detection algorithm.

This process is described in more details in Algorithm 1.

**Algorithm 1:**
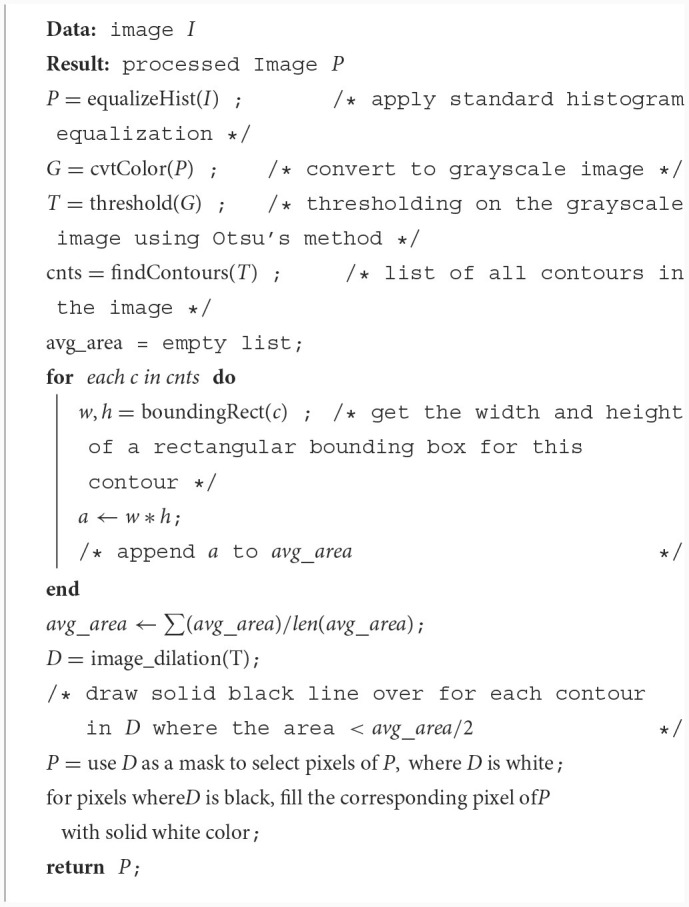
Background removal.

## 3 Results

### 3.1 Demographic data of FL patients

**Figure 5:**
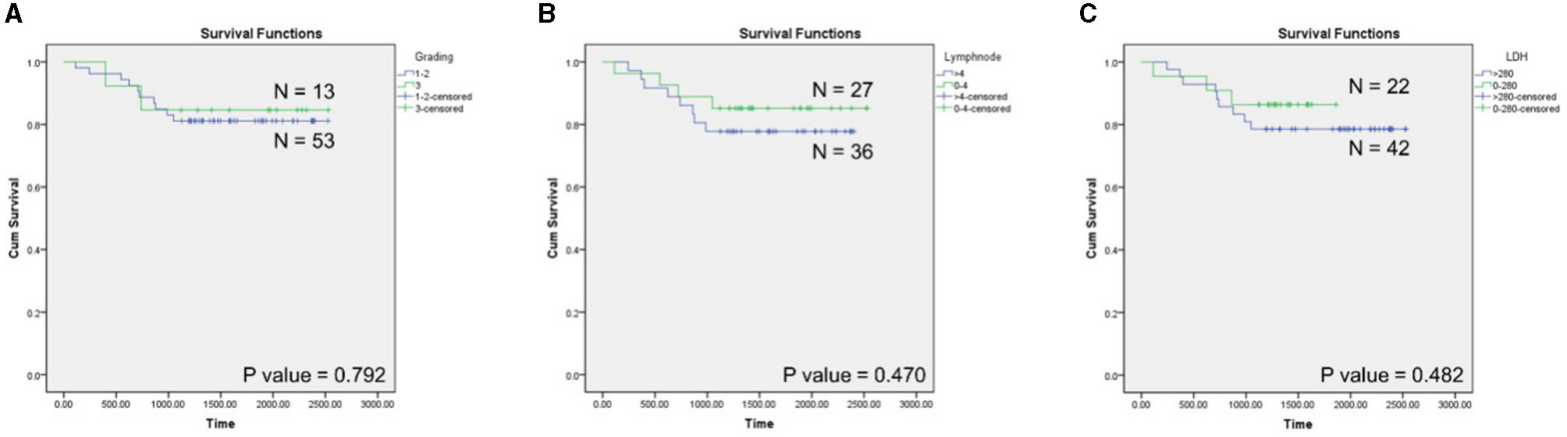
Kaplan–Meier analysis of FL patients. **(A)** Grading, **(B)** nodal areas involvement, and **(C)** serum lactate dehydrogenase.

Most of the patients were <62 years of age, and half of them were male and half were female (Table 1). Based on the WHO criteria, grade 1 has 0–5 CB/HPF, grade 2 has 6–15 CB/HPF, and grade 3 has more than 15 CB/HPF (3a-centrocytes present, 3b-solid sheets of CBs); most cases (70/87, 80.5%) were classified with grades 1 and 2; and only 17 cases (19.5%) were in grade 3 (either 3a or 3b) with grade 3B as around 3.4% of the total cases (Table 1). Grades 1, 2, and 3a are generally thought to be low-grade or slow growing, whereas grade 3b is likely to be treated as a high grade with a fast-growing phenotype. Notably, no significant correlation of clinicopathological parameters was observed (data not shown). Twelve patients with non-cancer-related death were excluded from the survival analysis. Hence, cumulative survival analysis of only 66, 63, and 66 cases were analyzed with grading, numbers of involved nodes, and serum lactate dehydrogenase level, respectively (Figure 5). High numbers of involving lymph nodes and high serum lactate dehydrogenase levels exhibited the trend of patient-short survival time correlation but without statistical significance.

### 3.2 The impact of preprocessing and background removal

The WSIs that we obtained come in two color variations: the first is the characteristic purple hue of the H&E stain and the second is pink. The pink color is from old glass slides that had been left in storage for a long time before getting digitized. Hematoxylin (the H in H&E) is less durable in the eosin, which has a pink color. Thus, glass slides that had been stored for a long time had mostly eosin and hence had a pink color. It is well-known from image processing that color variation as well as the brightness and contrast has a significant impact on any algorithm. Thus, histogram normalization (25) was considered as the preprocessing step. The model size was fixed at m for this experiment to minimize the training time. The result is shown in Table 3, where set 1 consisted of 445 patches obtained from the first round of labeling without any preprocessing. Set 2 consists of the entire dataset of 1,119 images and set 3 was the entire dataset with histogram normalization applied to the images before they enter model training. The result shows that the benefit of applying histogram normalization is inconclusive, and it slightly increases the value of mAP@0.5 and slightly decreases the value of mAP. Regardless of preprocessing, all three cases demonstrate relatively high value for mAP.

**Table 3:** Results of varying the preprocessing (histogram normalization or not), where the model size was fixed at YOLOv5m.

**Dataset**	**Precision**	**Recall**	**mAP@0.5**	**mAP**
Set 1 (445 images)	0.706	0.537	0.635	0.377
Set 2 (1,119 images)	0.623	0.585	0.643	0.381
Set 3 (1,119 images with histogram normalization)	0.698	0.619	0.659	0.377

### 3.3 The impact of different input and model sizes

**Figure 6:**
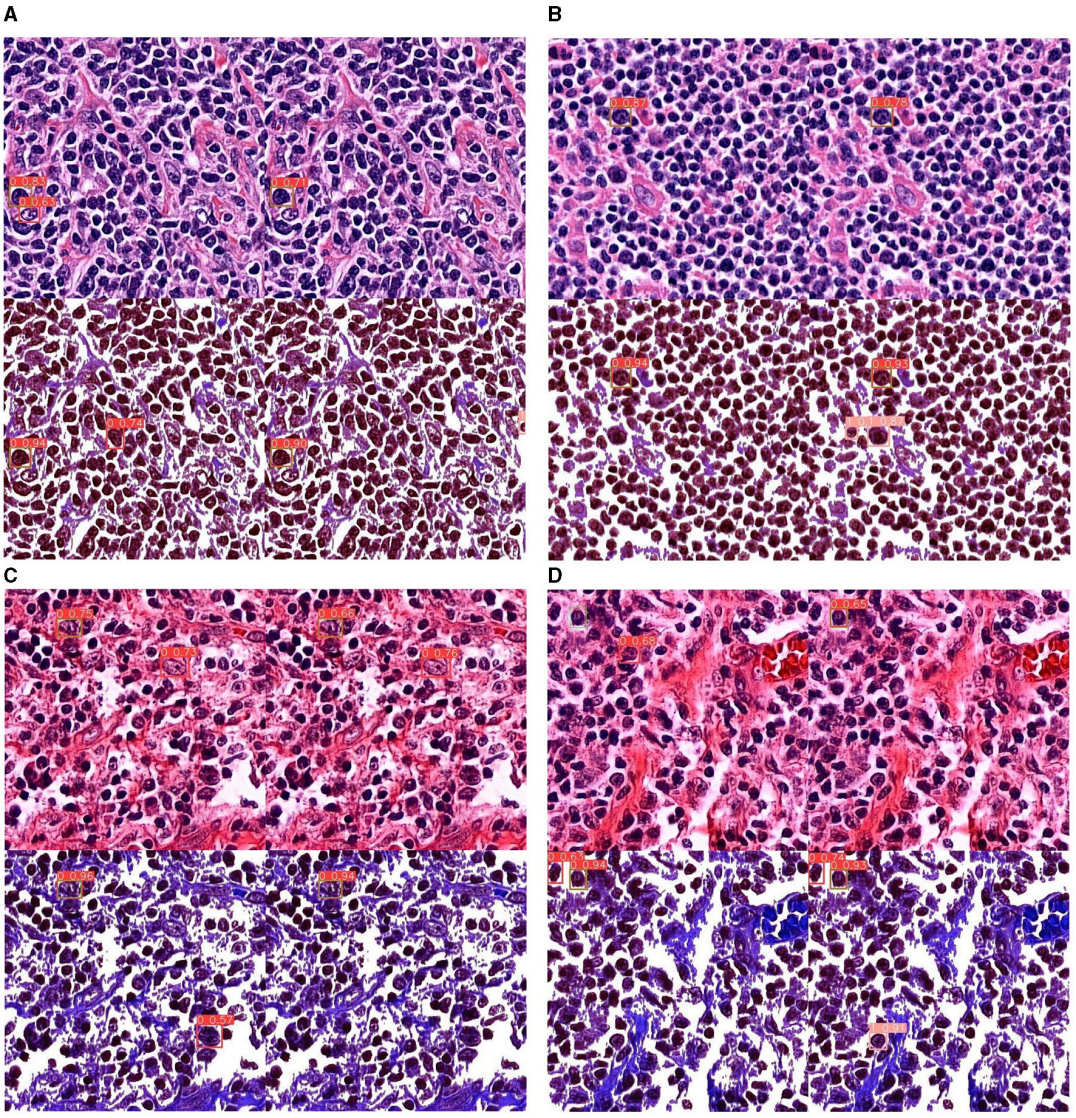
Detection results of all experiments on YOLOv5x6. **(A, B)** Detection results on purple-tinted WSI (newer slides), without and with background subtraction. **(C, D)** Detection results on pink-tinted WSI (older slides where discoloration had occurred), without and with background subtraction.

In this section, we experimented with different input image and model sizes to find the best configuration. Generally, deep learning tends to perform better with larger sized models. The biggest size available for YOLOv5 is x6. We tested the performance of this model size with different input image sizes: 512, 640, and 1,280 (the common practice is to resize input images to be squares). We also consider the m size model since smaller model are faster to run so if the performance gap is not too large, smaller models are preferred in practice. The result is shown in Table 4. Out of all the configurations, model size x6 with input size 512 has the best performance across all values. Larger input sizes did not perform very well, most likely because the HPF patches were originally obtained from WSIs at this resolution. Images for sizes 640 and 1,280 were obtained by up-sampling, which impacted the clarity of the images. The best possible values for mAP@0.5 achieved was 0.6708, which is a relatively high value for object detection. Figure 6 shows some examples of detection result. It can be seen that the dye has two tones: the purple ones on the top row and the pink ones on the bottom. This is because some of the WSIs were stored as glass slide for a long time and had only recently been digitized, leading to color change from purple to pink. In general, the model seems to perform better on purple-colored patches.

**Table 4:** The result of varying the model and input image sizes.

**Model size, input size**	**Precision**	**Recall**	**mAP@0.5**	**mAP**
YOLOv5m, 512	0.6234	0.5851	0.6425	0.3809
YOLOv5x6, 512	**0.6755**	**0.6098**	**0.6708**	**0.3812**
YOLOv5x6, 640	0.6617	0.5976	0.6445	0.3868
YOLOv5x6, 1,280	0.5387	0.5854	0.5698	0.3230

The bold font indicates the row with the best overall result.

From Table 5, the CB detection result on Experiment 1, the default CB dataset gives the highest evaluation result from YOLOv5x6 from using model achieved from Exp1-1, default CB dataset training. The best model achieved precision at 0.662, recall at 0.598, mAP at 0.5, IoU at 0.645, and overall mAP at 0.387. Where the CB and non-CB datasets show decrease values of evaluation metrics, the non-CBs instance may cause confusion to the model as it is very similar.

**Table 5:** CB detection results using experiment 1.

**Model**	**Method**	**Precision**	**Recall**	**mAP@0.5**	**mAP**
YOLOv5m	Exp1-1	0.623	0.585	0.643	0.381
YOLOv5m	Exp1-2	0.347	0.523	0.408	0.284
YOLOv5x6	Exp1-1	**0.662**	**0.598**	**0.645**	**0.387**
YOLOv5x6	Exp1-2	0.345	0.507	0.394	0.291

The bold font indicates the row with the best overall result.

Comparing to the CB detection result on Experiment 2, from Table 6, the preprocess CB dataset gives the highest evaluation result from YOLOv5m using model achieved from Exp2-1, preprocess CB dataset training. The best model achieved precision at 0.758, recall at 0.788, mAP at 0.5, IoU at 0.809, and overall mAP at 0.653. YOLOv5x6 evaluation is more average in all metrics as it achieved precision at 0.808, recall at 0.776, mAP at 0.5, IoU at 0.800, and overall mAP at 0.647. The CB and non-CB datasets show slightly decreased values to the non-CBs instance, which may cause confusion to the model as it is very similar. Moreover, the preprocessing CB dataset can greatly enhance the evaluation value though they are trained in the same manner. The background removal of other fluid other than cells can immensely impact detection training.

**Table 6:** CB detection results using experiment 2.

**Model**	**Method**	**Precision**	**Recall**	**mAP@0.5**	**mAP**
YOLOv5m	Exp2-1	0.758	0.788	0.809	0.653
YOLOv5m	Exp2-2	0.801	0.722	0.739	0.618
YOLOv5x6	Exp2-1	**0.808**	**0.776**	**0.800**	**0.647**
YOLOv5x6	Exp2-2	0.837	0.740	0.747	0.633

The bold font indicates the row with the best overall result.

**Figure 7:**
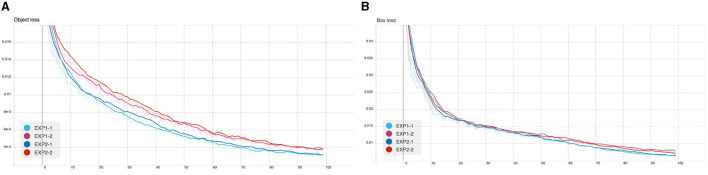
CB detection model training: **(A)** object loss and **(B)** box losses.

**Figure 8:**
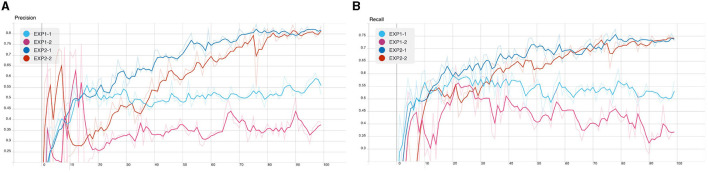
CB detection model training: **(A)** precision and **(B)** recall.

**Figure 9:**
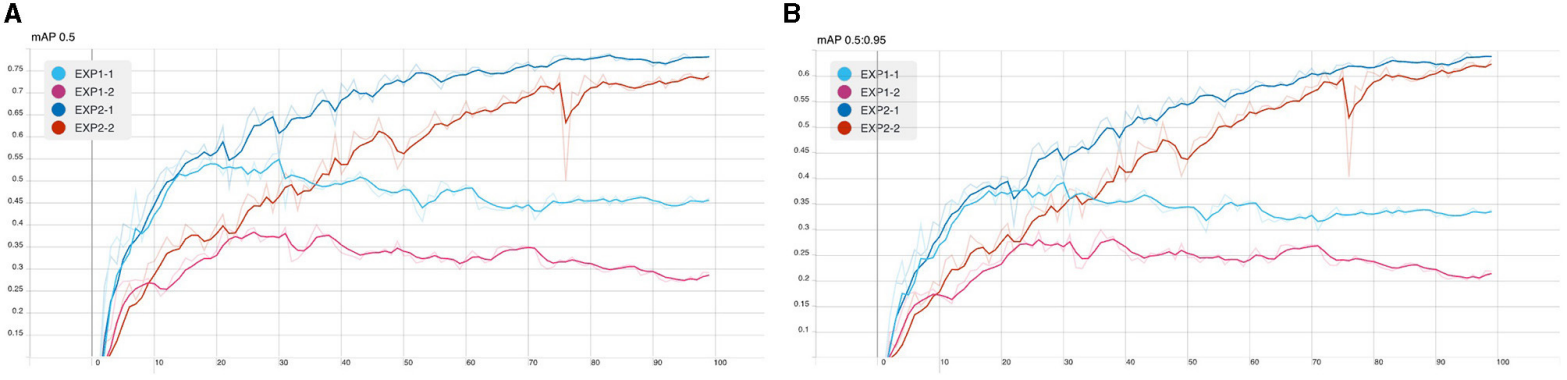
CB detection model training: **(A)** mAP@0.5 and **(B)** mAP overall.

From the following experiments, the CB object detection model training using YOLOv5 has been trained with defaults training hyperparameter in all experiments for 100 epochs. Figures 7–9 shows training evaluation of CB detection in each experiment setup comparing training on default CB dataset and preprocess CB dataset. The YOLOv5x6 model using preprocess CB dataset training demonstrated the best model as its training losses and evaluation metrics, such as precision, recall, and mAP values, lead the other setups.

The CB detection models are used for detecting CB on test dataset; Figure 6 shows four sample of results from each experiment that effect CB detection. As an image contains four results of each experiment, where:

- Top left of the image represents detection result from default CB dataset.
- Top right of the image represents detection result from default CB and non-CB datasets.
- Bottom left of the image represents detection result from preprocess CB dataset.
- Bottom right of the image represents detection result from preprocess CB and non-CB datasets.

The preprocess CB creates a clear cell shape and higher contrast images as shown in Figure 6, which it can significantly enhance confidence of detection score. The red boxes indicate prediction of CB, pink boxes indicate non-CB, and green boxes indicate ground truth of CBs instances. Moreover, the results show less false positive detection in preprocess CB and non-CB datasets results. Even though the preprocess might not perform well in very low contrast images or low component images, where the method can reduce the features of CB cell where the inside is too hollow, it leads to creating true positive results. This method may also impact on other cells where it might represent similar characteristics as CB, as the inside of the cell is also having hollow space, creating false positive results. Overall, the YOLOv5x6 with preprocess CB and non-CB datasets training yield the best results, as it can increase confidential of detection and reduce false positive rate.

## 4 Discussion

The proposed method works on the level of individual patches. Specifically, our method cuts a WSI into individual patches of the same size, at the native resolution, and then applies the CB detection algorithm to each patch. This approach is not computationally efficient since a WSI can have tens of thousands of patches, most of which are not inspected by the pathologist reading the slide. Typically, a pathologist would inspect the WSI at a lower magnification, choose some regions of interest (ROIs), and then perform high power field reading only on patches in the ROIs. Cells that may have the morphological characteristics of a CB are not counted if they are not inside one of the ROIs. At present, the proposed method has to rely on pathologists choosing the ROIs manually, and then offload the high power field reading to the detection algorithm. While this helps reduce the workload, it still requires experienced pathologists to appropriately choose the ROIs. We plan to address this disadvantage in the future study, where the currently proposed algorithms would receive the ROIs information from another algorithm that operates at a lower magnification level looking at more global features of the WSI. The goal is to make the reading entirely automated.

## 5 Limitation

The proposed method needs global ROIs to be provided by pathologists and it works only for H&E stain, preferably recent such that no color fading had not yet occurred. The model was only tested on WSIs that are digitized in the .mrxs file type that can be read by the Python Openslide library. Running the model can be computationally expensive and requires more resources than a lab may have available.

## 6 Conclusion

We present an experimental study for the automatic detection and counting of centroblast cells in whole slide images. The method consists of applying a deep-learning based object detection architecture and algorithm, on top of which we proposed improvements to detect individual centroblast cells from patches of WSI images, from which the number of CB cells can be counted and thus obtain the grading result. We performed ablative studies of the different configurations of the object detection pipeline. Two key steps in this proposed pipeline is the hard-negative mining steps (Figure 7) and background removal step (Algorithm 1). Using the hard negative mining but not the background removal step in the pipeline gave the best result in terms of recall value. Using both the hard negative mining steps and the background removal step gave the best overall result in terms of the mAP values. The proposed improvements boosted the detection result significantly from the standard application of object detection, as can be seen by comparing the results in Tables 5, 6.

## Data availability statement

The original contributions presented in the study are included in the article/supplementary material, further inquiries can be directed to the corresponding authors.

## Ethics statement

The studies involving humans were approved by Siriraj Institutional Review Board (SIRB) (MU-MOU CoA No. 973/2020). The studies were conducted in accordance with the local legislation and institutional requirements. The human samples used in this study were acquired from a by- product of routine care or industry. Written informed consent for participation was not required from the participants or the participants' legal guardians/next of kin in accordance with the national legislation and institutional requirements.

## Author contributions

SY: Formal analysis, Investigation, Methodology, Software, Validation, Visualization, Writing – original draft, Writing – review & editing. PB: Data curation, Writing – review & editing. SS: Data curation, Writing – review & editing. PT: Conceptualization, Data curation, Writing – review & editing. KC: Conceptualization, Data curation, Writing – review & editing. AP: Data curation, Writing – review & editing. NA: Data curation, Writing – review & editing. NH: Conceptualization, Formal analysis, Investigation, Methodology, Software, Validation, Visualization, Writing – review & editing. CT: Conceptualization, Data curation, Formal analysis, Funding acquisition, Methodology, Project administration, Validation, Visualization, Writing – review & editing.
